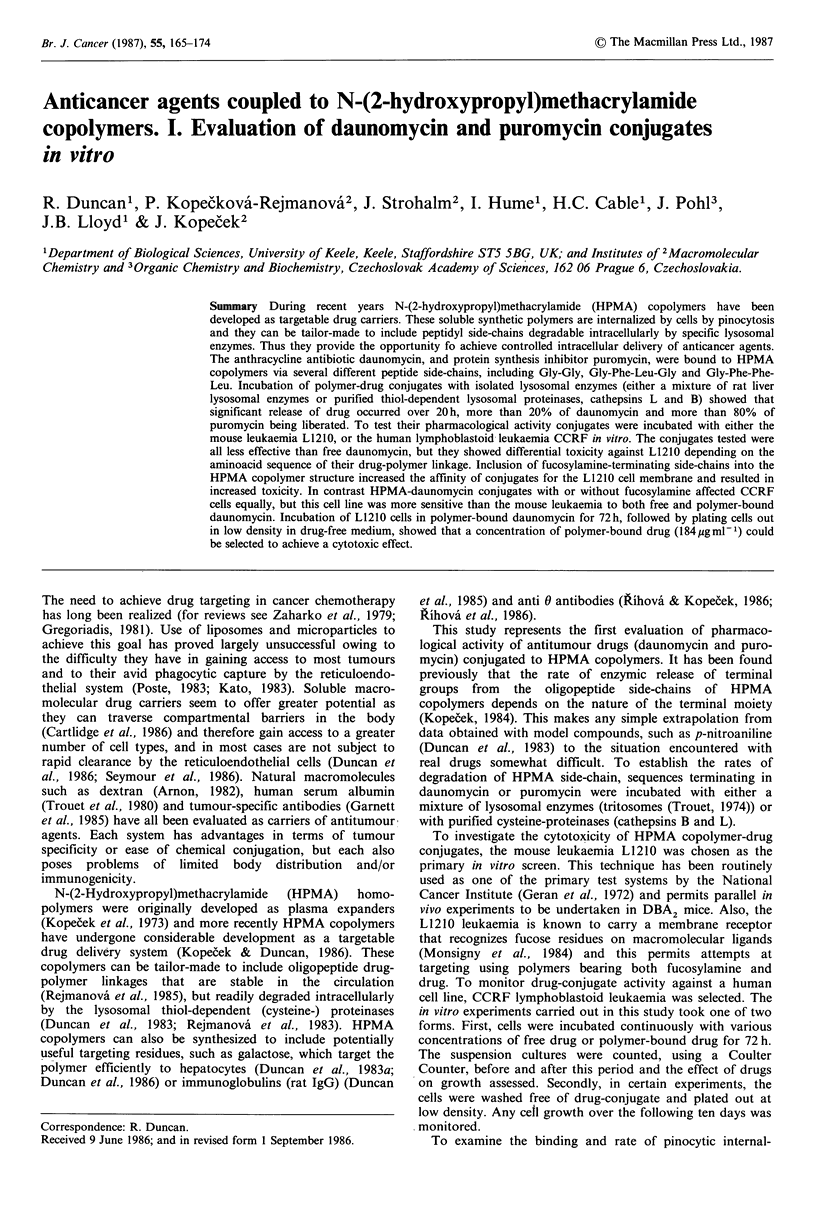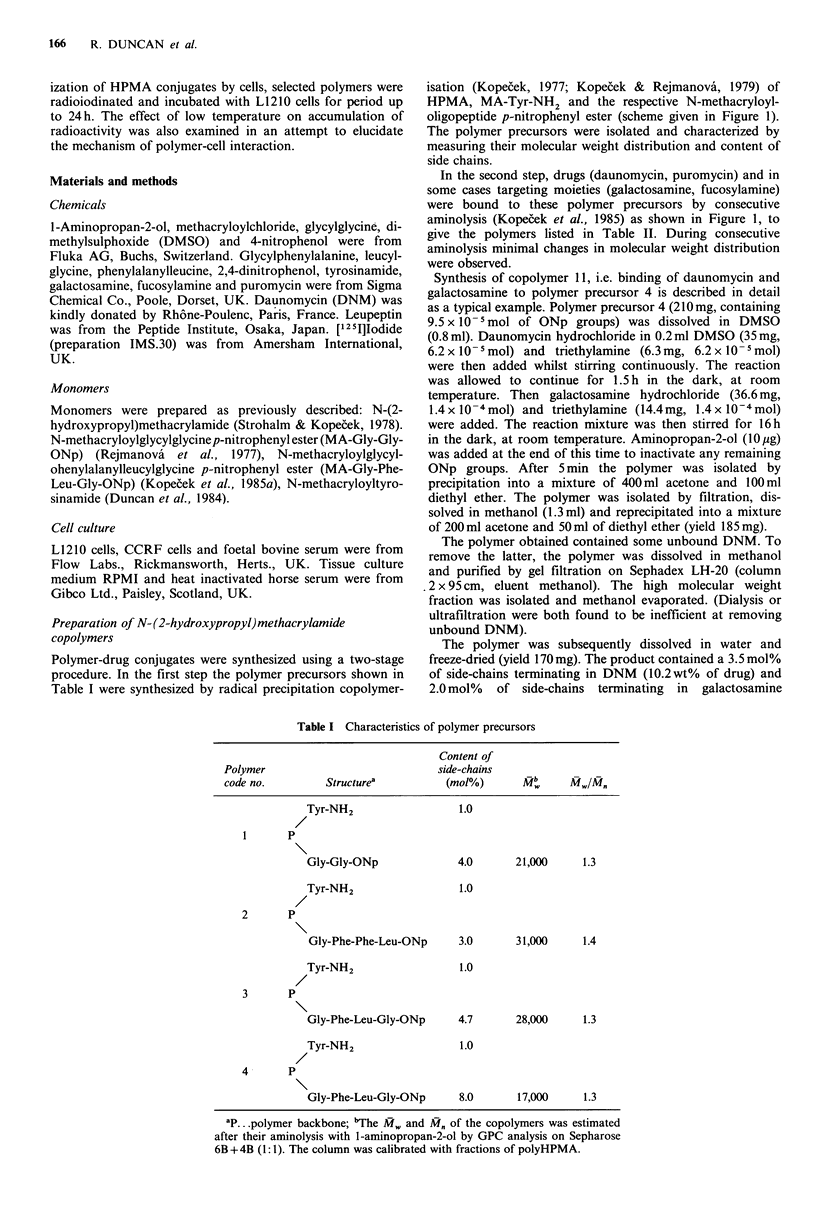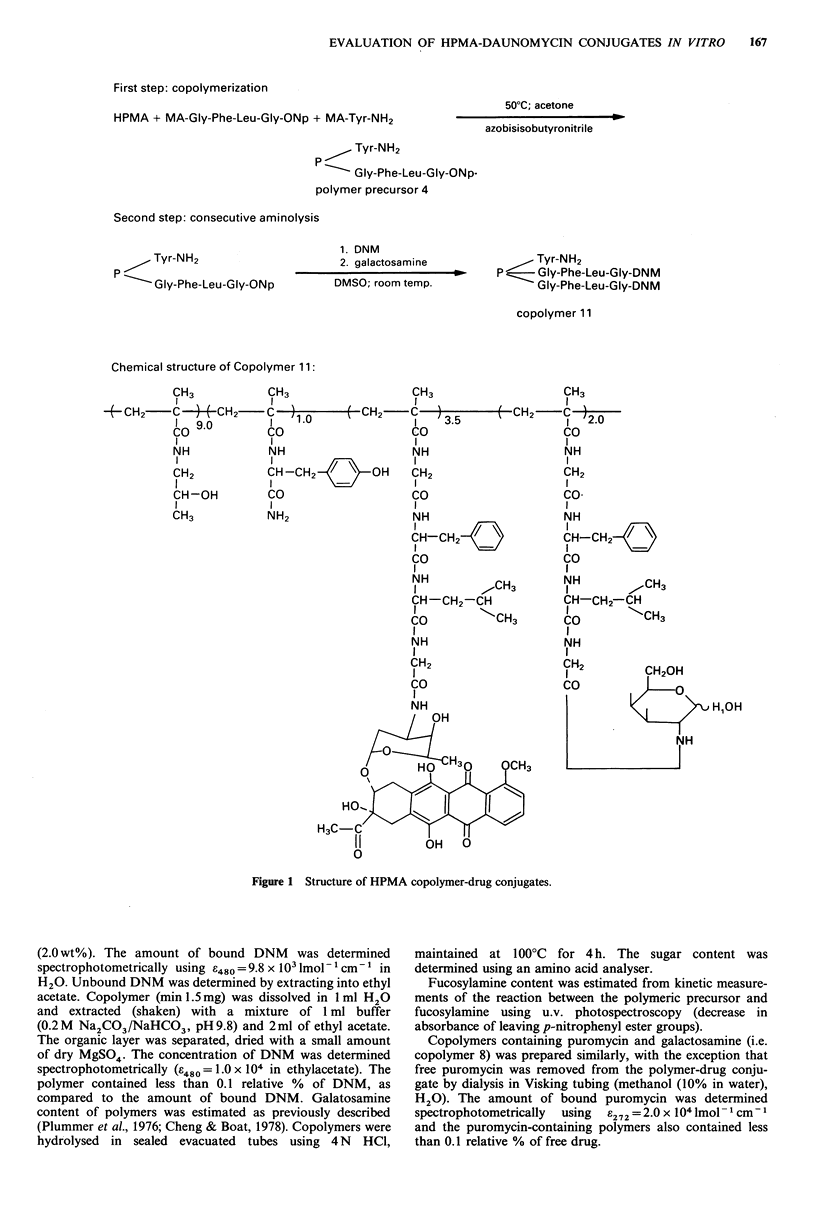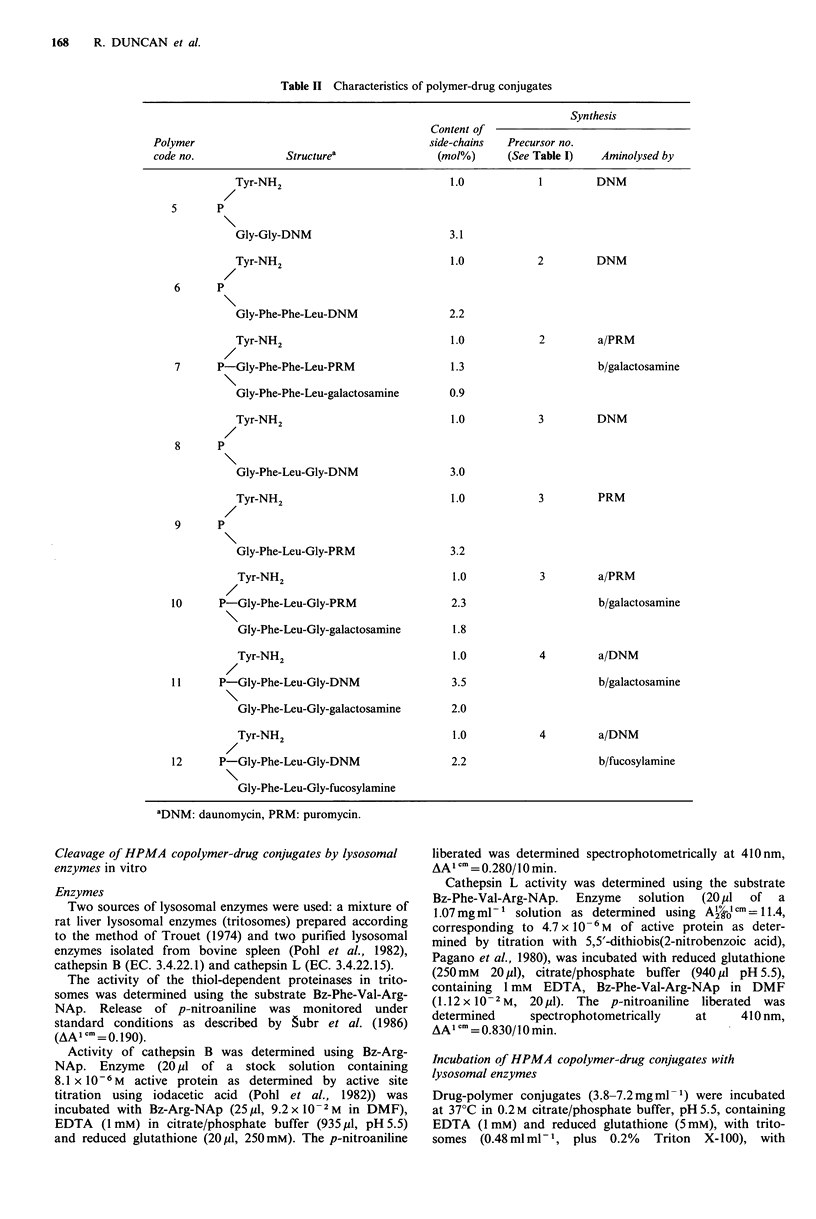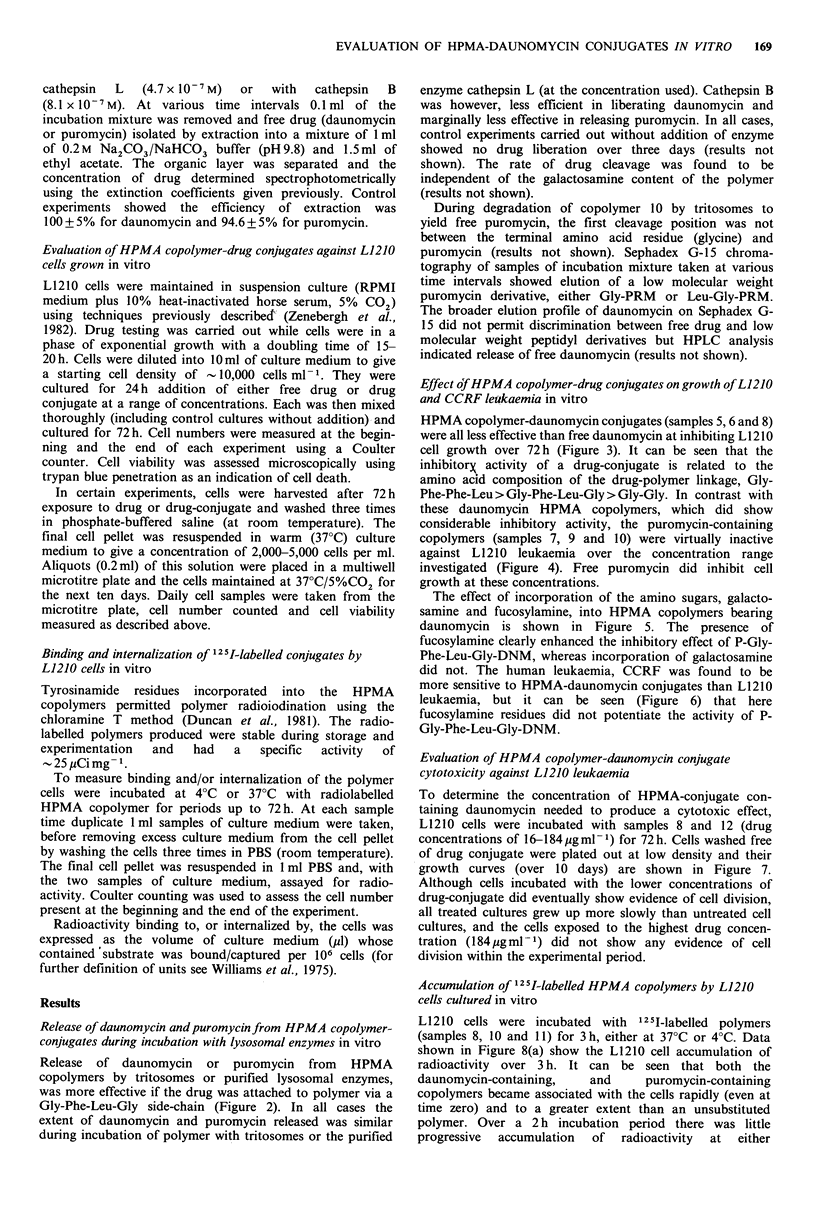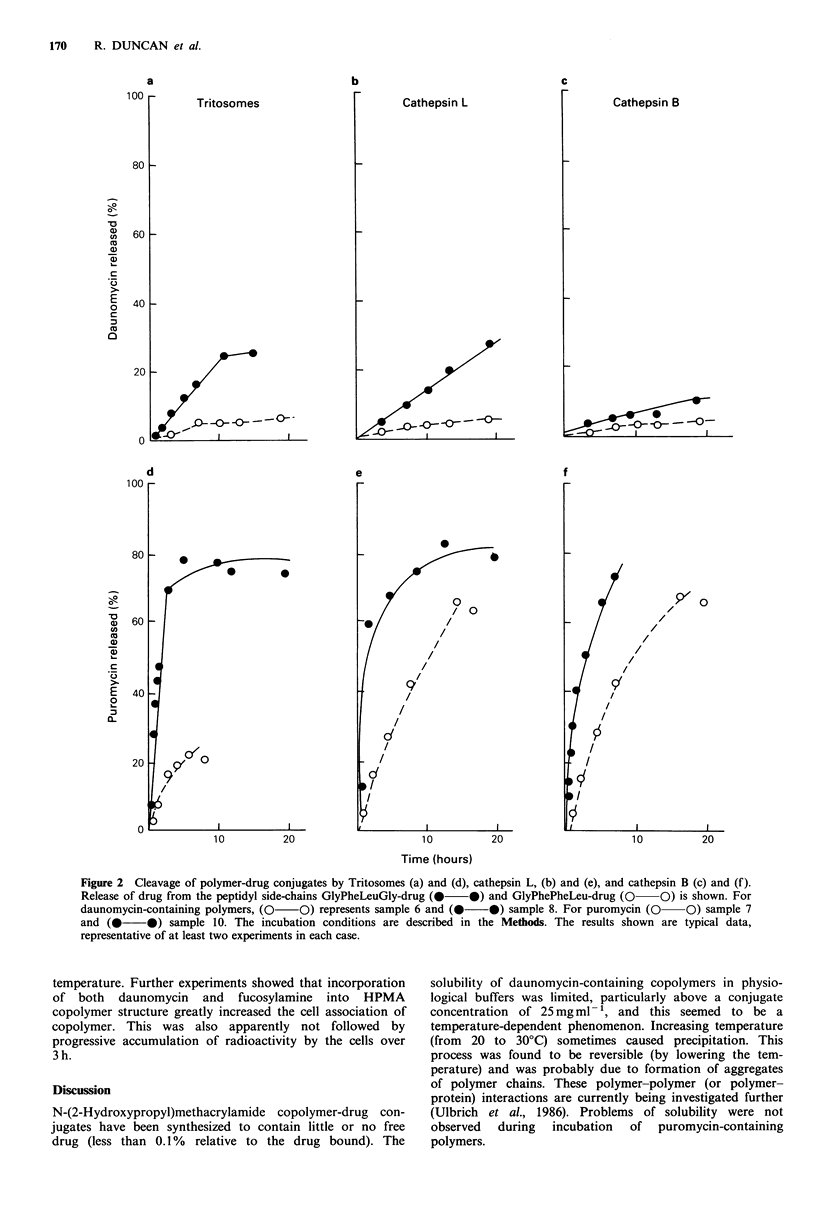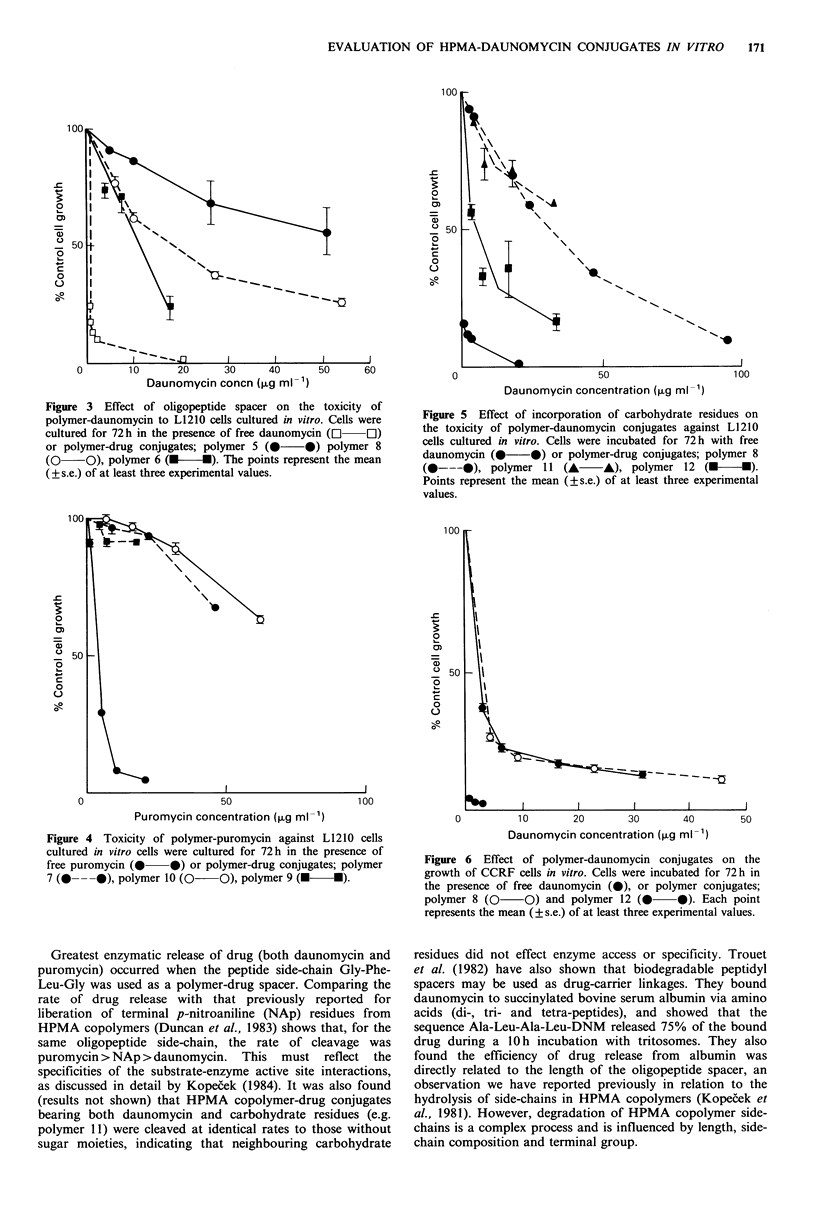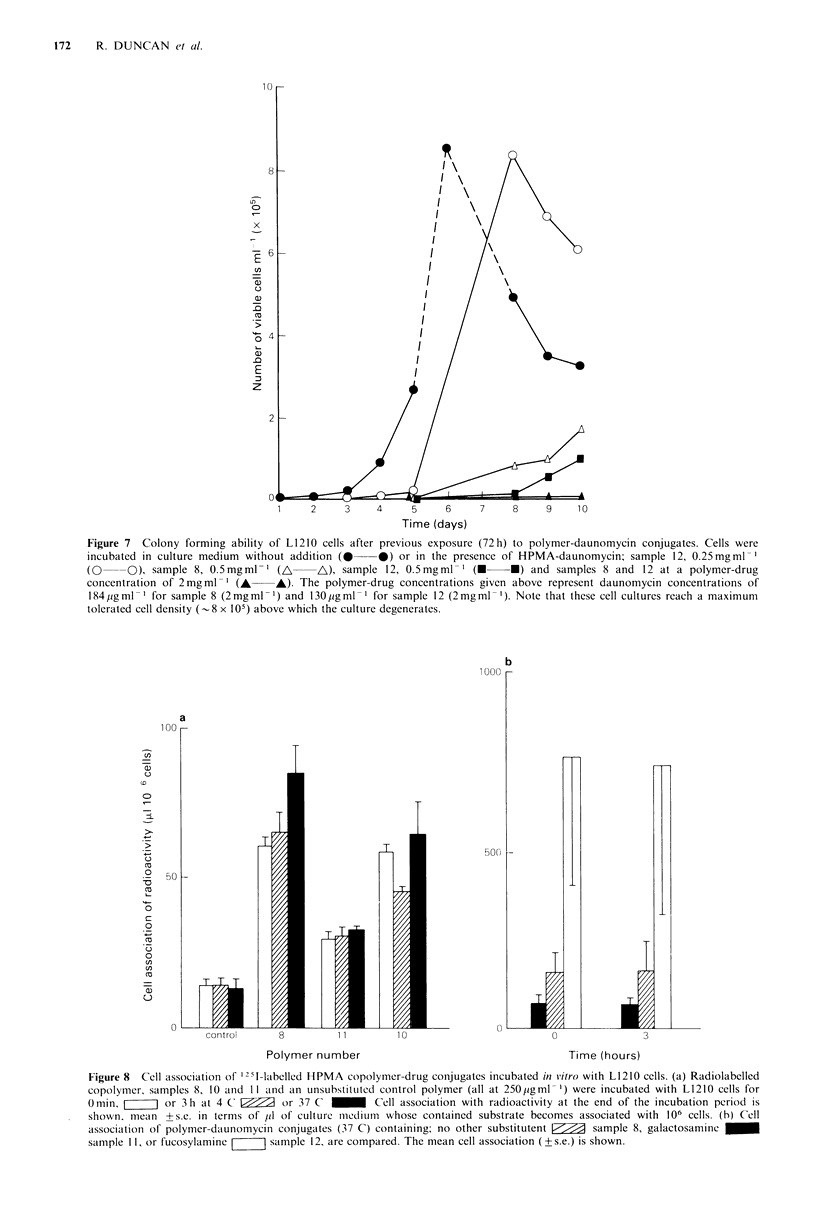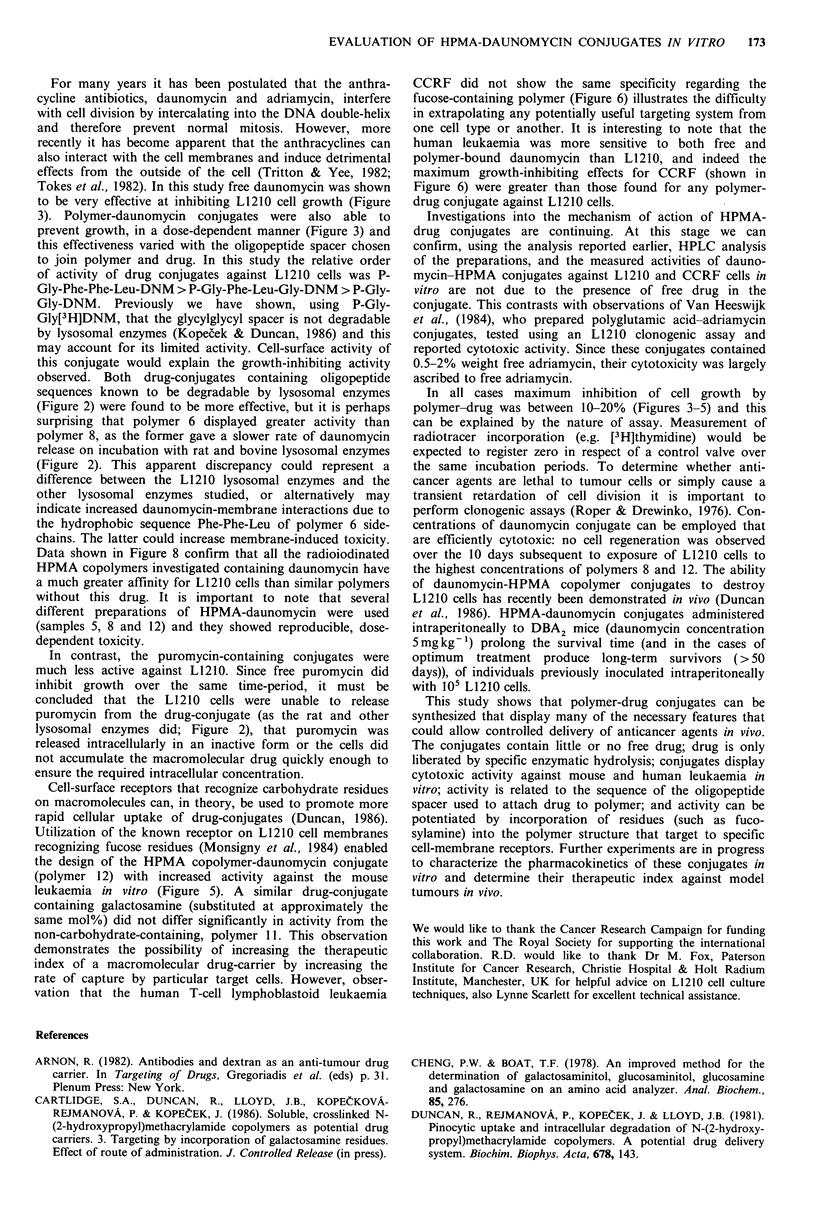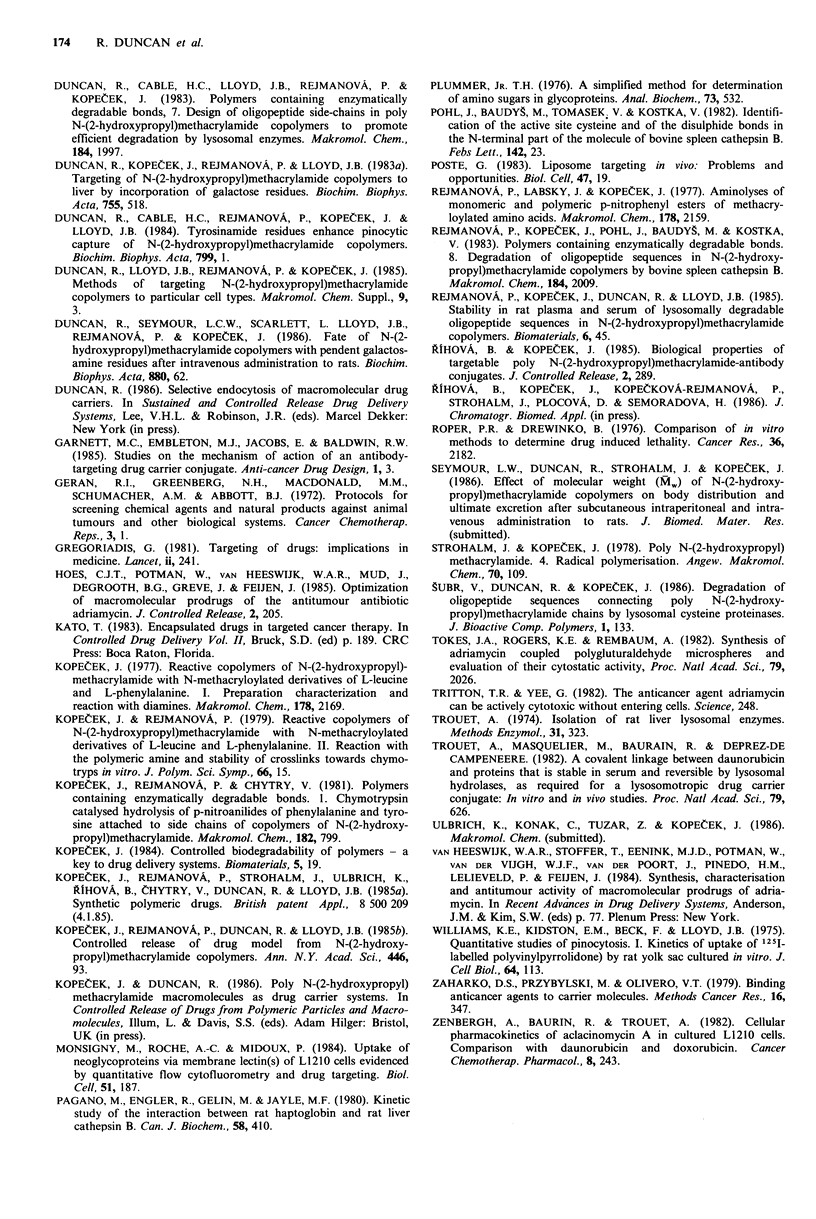# Anticancer agents coupled to N-(2-hydroxypropyl)methacrylamide copolymers. I. Evaluation of daunomycin and puromycin conjugates in vitro.

**DOI:** 10.1038/bjc.1987.33

**Published:** 1987-02

**Authors:** R. Duncan, P. Kopecková-Rejmanová, J. Strohalm, I. Hume, H. C. Cable, J. Pohl, J. B. Lloyd, J. Kopecek

## Abstract

During recent years N-(2-hydroxypropyl)methacrylamide (HPMA) copolymers have been developed as targetable drug carriers. These soluble synthetic polymers are internalized by cells by pinocytosis and they can be tailor-made to include peptidyl side-chains degradable intracellularly by specific lysosomal enzymes. Thus they provide the opportunity fo achieve controlled intracellular delivery of anticancer agents. The anthracycline antibiotic daunomycin, and protein synthesis inhibitor puromycin, were bound to HPMA copolymers via several different peptide side-chains, including Gly-Gly, Gly-Phe-Leu-Gly and Gly-Phe-Phe-Leu. Incubation of polymer-drug conjugates with isolated lysosomal enzymes (either a mixture of rat liver lysosomal enzymes or purified thiol-dependent lysosomal proteinases, cathepsins L and B) showed that significant release of drug occurred over 20 h, more than 20% of daunomycin and more than 80% of puromycin being liberated. To test their pharmacological activity conjugates were incubated with either the mouse leukaemia L1210, or the human lymphoblastoid leukaemia CCRF in vitro. The conjugates tested were all less effective than free daunomycin, but they showed differential toxicity against L1210 depending on the aminoacid sequence of their drug-polymer linkage. Inclusion of fucosylamine-terminating side-chains into the HPMA copolymer structure increased the affinity of conjugates for the L1210 cell membrane and resulted in increased toxicity. In contrast HPMA-daunomycin conjugates with or without fucosylamine affected CCRF cells equally, but this cell line was more sensitive than the mouse leukaemia to both free and polymer-bound daunomycin. Incubation of L1210 cells in polymer-bound daunomycin for 72 h, followed by plating cells out in low density in drug-free medium, showed that a concentration of polymer-bound drug (184 micrograms ml-1) could be selected to achieve a cytotoxic effect.


*Br. J. Cancer* (1987), **55**, 165–174 © The Macmillan Press Ltd., 1987

### Anticancer agents coupled to N-(2-hydroxypropyl)methacrylamide copolymers. I. Evaluation of daunomycin and puromycin conjugates *in vitro*

R. Duncan[1], P. Kopečková-Rejmanová[2], J. Strohalm[2], I. Hume[1], H.C. Cable[1], J. Pohl[3], J.B. Lloyd[1] & J. Kopeček[2]

[1]*Department of Biological Sciences, University of Keele, Keele, Staffordshire ST5 5BG, UK; and Institutes of* [2]*Macromolecular Chemistry and* [3]*Organic Chemistry and Biochemistry, Czechoslovak Academy of Sciences, 162 06 Prague 6, Czechoslovakia.*

**Summary** During recent years N-(2-hydroxypropyl)methacrylamide (HPMA) copolymers have been developed as targetable drug carriers. These soluble synthetic polymers are internalized by cells by pinocytosis and they can be tailor-made to include peptidyl side-chains degradable intracellularly by specific lysosomal enzymes. Thus they provide the opportunity fo achieve controlled intracellular delivery of anticancer agents. The anthracycline antibiotic daunomycin, and protein synthesis inhibitor puromycin, were bound to HPMA copolymers via several different peptide side-chains, including Gly-Gly, Gly-Phe-Leu-Gly and Gly-Phe-Phe-Leu. Incubation of polymer-drug conjugates with isolated lysosomal enzymes (either a mixture of rat liver lysosomal enzymes or purified thiol-dependent lysosomal proteinases, cathepsins L and B) showed that significant release of drug occurred over 20 h, more than 20% of daunomycin and more than 80% of puromycin being liberated. To test their pharmacological activity conjugates were incubated with either the mouse leukaemia L1210, or the human lymphoblastoid leukaemia CCRF *in vitro*. The conjugates tested were all less effective than free daunomycin, but they showed differential toxicity against L1210 depending on the aminoacid sequence of their drug-polymer linkage. Inclusion of fucosylamine-terminating side-chains into the HPMA copolymer structure increased the affinity of conjugates for the L1210 cell membrane and resulted in increased toxicity. In contrast HPMA-daunomycin conjugates with or without fucosylamine affected CCRF cells equally, but this cell line was more sensitive than the mouse leukaemia to both free and polymer-bound daunomycin. Incubation of L1210 cells in polymer-bound daunomycin for 72 h, followed by plating cells out in low density in drug-free medium, showed that a concentration of polymer-bound drug (184 $\mu g\,ml^{-1}$) could be selected to achieve a cytotoxic effect.

The need to achieve drug targeting in cancer chemotherapy has long been realized (for reviews see Zaharko *et al.*, 1979; Gregoriadis, 1981). Use of liposomes and microparticles to achieve this goal has proved largely unsuccessful owing to the difficulty they have in gaining access to most tumours and to their avid phagocytic capture by the reticuloendothelial system (Poste, 1983; Kato, 1983). Soluble macromolecular drug carriers seem to offer greater potential as they can traverse compartmental barriers in the body (Cartlidge *et al.*, 1986) and therefore gain access to a greater number of cell types, and in most cases are not subject to rapid clearance by the reticuloendothelial cells (Duncan *et al.*, 1986; Seymour *et al.*, 1986). Natural macromolecules such as dextran (Arnon, 1982), human serum albumin (Trouet *et al.*, 1980) and tumour-specific antibodies (Garnett *et al.*, 1985) have all been evaluated as carriers of antitumour agents. Each system has advantages in terms of tumour specificity or ease of chemical conjugation, but each also poses problems of limited body distribution and/or immunogenicity.

N-(2-Hydroxypropyl)methacrylamide (HPMA) homopolymers were originally developed as plasma expanders (Kopeček *et al.*, 1973) and more recently HPMA copolymers have undergone considerable development as a targetable drug delivery system (Kopeček & Duncan, 1986). These copolymers can be tailor-made to include oligopeptide drug-polymer linkages that are stable in the circulation (Rejmanová *et al.*, 1985), but readily degraded intracellularly by the lysosomal thiol-dependent (cysteine-) proteinases (Duncan *et al.*, 1983; Rejmanová *et al.*, 1983). HPMA copolymers can also be synthesized to include potentially useful targeting residues, such as galactose, which target the polymer efficiently to hepatocytes (Duncan *et al.*, 1983*a*; Duncan *et al.*, 1986) or immunoglobulins (rat IgG) (Duncan *et al.*, 1985) and anti $\theta$ antibodies (Říhová & Kopeček, 1986; Říhová *et al.*, 1986).

This study represents the first evaluation of pharmacological activity of antitumour drugs (daunomycin and puromycin) conjugated to HPMA copolymers. It has been found previously that the rate of enzymic release of terminal groups from the oligopeptide side-chains of HPMA copolymers depends on the nature of the terminal moiety (Kopeček, 1984). This makes any simple extrapolation from data obtained with model compounds, such as *p*-nitroaniline (Duncan *et al.*, 1983) to the situation encountered with real drugs somewhat difficult. To establish the rates of degradation of HPMA side-chain, sequences terminating in daunomycin or puromycin were incubated with either a mixture of lysosomal enzymes (tritosomes (Trouet, 1974)) or with purified cysteine-proteinases (cathepsins B and L).

To investigate the cytotoxicity of HPMA copolymer-drug conjugates, the mouse leukaemia L1210 was chosen as the primary *in vitro* screen. This technique has been routinely used as one of the primary test systems by the National Cancer Institute (Geran *et al.*, 1972) and permits parallel *in vivo* experiments to be undertaken in $DBA_2$ mice. Also, the L1210 leukaemia is known to carry a membrane receptor that recognizes fucose residues on macromolecular ligands (Monsigny *et al.*, 1984) and this permits attempts at targeting using polymers bearing both fucosylamine and drug. To monitor drug-conjugate activity against a human cell line, CCRF lymphoblastoid leukaemia was selected. The *in vitro* experiments carried out in this study took one of two forms. First, cells were incubated continuously with various concentrations of free drug or polymer-bound drug for 72 h. The suspension cultures were counted, using a Coulter Counter, before and after this period and the effect of drugs on growth assessed. Secondly, in certain experiments, the cells were washed free of drug-conjugate and plated out at low density. Any cell growth over the following ten days was monitored.

To examine the binding and rate of pinocytic internal-

Correspondence: R. Duncan.
Received 9 June 1986; and in revised form 1 September 1986.

ization of HPMA conjugates by cells, selected polymers were radioiodinated and incubated with L1210 cells for period up to 24 h. The effect of low temperature on accumulation of radioactivity was also examined in an attempt to elucidate the mechanism of polymer-cell interaction.

### Materials and methods

### *Chemicals*

1-Aminopropan-2-ol, methacryloylchloride, glycylglycine, dimethylsulphoxide (DMSO) and 4-nitrophenol were from Fluka AG, Buchs, Switzerland. Glycylphenylalanine, leucylglycine, phenylalanylleucine, 2,4-dinitrophenol, tyrosinamide, galactosamine, fucosylamine and puromycin were from Sigma Chemical Co., Poole, Dorset, UK. Daunomycin (DNM) was kindly donated by Rhône-Poulenc, Paris, France. Leupeptin was from the Peptide Institute, Osaka, Japan. [$^{125}$I]Iodide (preparation IMS.30) was from Amersham International, UK.

### *Monomers*

Monomers were prepared as previously described: N-(2-hydroxypropyl)methacrylamide (Strohalm & Kopeček, 1978). N-methacryloylglycylglycine *p*-nitrophenyl ester (MA-Gly-Gly-ONp) (Rejmanová *et al.*, 1977), N-methacryloylglycylohenylalanylleucylglycine *p*-nitrophenyl ester (MA-Gly-Phe-Leu-Gly-ONp) (Kopeček *et al.*, 1985*a*), N-methacryloyltyrosinamide (Duncan *et al.*, 1984).

### *Cell culture*

L1210 cells, CCRF cells and foetal bovine serum were from Flow Labs., Rickmansworth, Herts., UK. Tissue culture medium RPMI and heat inactivated horse serum were from Gibco Ltd., Paisley, Scotland, UK.

### *Preparation of N-(2-hydroxypropyl)methacrylamide copolymers*

Polymer-drug conjugates were synthesized using a two-stage procedure. In the first step the polymer precursors shown in Table I were synthesized by radical precipitation copolymerisation (Kopeček, 1977; Kopeček & Rejmanová, 1979) of HPMA, MA-Tyr-NH$_2$ and the respective N-methacryloyl-oligopeptide *p*-nitrophenyl ester (scheme given in Figure 1). The polymer precursors were isolated and characterized by measuring their molecular weight distribution and content of side chains.

In the second step, drugs (daunomycin, puromycin) and in some cases targeting moieties (galactosamine, fucosylamine) were bound to these polymer precursors by consecutive aminolysis (Kopeček *et al.*, 1985) as shown in Figure 1, to give the polymers listed in Table II. During consecutive aminolysis minimal changes in molecular weight distribution were observed.

Synthesis of copolymer 11, i.e. binding of daunomycin and galactosamine to polymer precursor 4 is described in detail as a typical example. Polymer precursor 4 (210 mg, containing $9.5 \times 10^{-5}$ mol of ONp groups) was dissolved in DMSO (0.8 ml). Daunomycin hydrochloride in 0.2 ml DMSO (35 mg, $6.2 \times 10^{-5}$ mol) and triethylamine (6.3 mg, $6.2 \times 10^{-5}$ mol) were then added whilst stirring continuously. The reaction was allowed to continue for 1.5 h in the dark, at room temperature. Then galactosamine hydrochloride (36.6 mg, $1.4 \times 10^{-4}$ mol) and triethylamine (14.4 mg, $1.4 \times 10^{-4}$ mol) were added. The reaction mixture was then stirred for 16 h in the dark, at room temperature. Aminopropan-2-ol (10 $\mu$g) was added at the end of this time to inactivate any remaining ONp groups. After 5 min the polymer was isolated by precipitation into a mixture of 400 ml acetone and 100 ml diethyl ether. The polymer was isolated by filtration, dissolved in methanol (1.3 ml) and reprecipitated into a mixture of 200 ml acetone and 50 ml of diethyl ether (yield 185 mg).

The polymer obtained contained some unbound DNM. To remove the latter, the polymer was dissolved in methanol and purified by gel filtration on Sephadex LH-20 (column $2 \times 95$ cm, eluent methanol). The high molecular weight fraction was isolated and methanol evaporated. (Dialysis or ultrafiltration were both found to be inefficient at removing unbound DNM).

The polymer was subsequently dissolved in water and freeze-dried (yield 170 mg). The product contained a 3.5 mol% of side-chains terminating in DNM (10.2 wt% of drug) and 2.0 mol% of side-chains terminating in galactosamine

**Table I** Characteristics of polymer precursors

| Polymer code no. | Structure[a] | Content of side-chains (mol%) | $\bar{M}_w^b$ | $\bar{M}_w/\bar{M}_n$ |
|---|---|---|---|---|
| | Tyr-NH$_2$ | 1.0 | | |
| 1 | P | | | |
| | Gly-Gly-ONp | 4.0 | 21,000 | 1.3 |
| | Tyr-NH$_2$ | 1.0 | | |
| 2 | P | | | |
| | Gly-Phe-Phe-Leu-ONp | 3.0 | 31,000 | 1.4 |
| | Tyr-NH$_2$ | 1.0 | | |
| 3 | P | | | |
| | Gly-Phe-Leu-Gly-ONp | 4.7 | 28,000 | 1.3 |
| | Tyr-NH$_2$ | 1.0 | | |
| 4 | P | | | |
| | Gly-Phe-Leu-Gly-ONp | 8.0 | 17,000 | 1.3 |

[a]P...polymer backbone; [b]The $\bar{M}_w$ and $\bar{M}_n$ of the copolymers was estimated after their aminolysis with 1-aminopropan-2-ol by GPC analysis on Sepharose 6B+4B (1:1). The column was calibrated with fractions of polyHPMA.

Figure 1 Structure of HPMA copolymer-drug conjugates.

(2.0 wt%). The amount of bound DNM was determined spectrophotometrically using $\varepsilon_{480} = 9.8 \times 10^3 \, l mol^{-1} cm^{-1}$ in $H_2O$. Unbound DNM was determined by extracting into ethyl acetate. Copolymer (min 1.5 mg) was dissolved in 1 ml $H_2O$ and extracted (shaken) with a mixture of 1 ml buffer (0.2 M $Na_2CO_3/NaHCO_3$, pH 9.8) and 2 ml of ethyl acetate. The organic layer was separated, dried with a small amount of dry $MgSO_4$. The concentration of DNM was determined spectrophotometrically ($\varepsilon_{480} = 1.0 \times 10^4$ in ethylacetate). The polymer contained less than 0.1 relative % of DNM, as compared to the amount of bound DNM. Galatosamine content of polymers was estimated as previously described (Plummer *et al.*, 1976; Cheng & Boat, 1978). Copolymers were hydrolysed in sealed evacuated tubes using 4 N HCl, maintained at 100°C for 4 h. The sugar content was determined using an amino acid analyser.

Fucosylamine content was estimated from kinetic measurements of the reaction between the polymeric precursor and fucosylamine using u.v. photospectroscopy (decrease in absorbance of leaving *p*-nitrophenyl ester groups).

Copolymers containing puromycin and galactosamine (i.e. copolymer 8) was prepared similarly, with the exception that free puromycin was removed from the polymer-drug conjugate by dialysis in Visking tubing (methanol (10% in water), $H_2O$). The amount of bound puromycin was determined spectrophotometrically using $\varepsilon_{272} = 2.0 \times 10^4 \, l mol^{-1} cm^{-1}$ and the puromycin-containing polymers also contained less than 0.1 relative % of free drug.

**Table II** Characteristics of polymer-drug conjugates

| Polymer code no. | Structure[a] | Content of side-chains (mol%) | Synthesis: Precursor no. (See **Table I**) | Synthesis: Aminolysed by |
|---|---|---|---|---|
| | Tyr-$NH_2$ | 1.0 | 1 | DNM |
| 5 | P | | | |
| | Gly-Gly-DNM | 3.1 | | |
| | Tyr-$NH_2$ | 1.0 | 2 | DNM |
| 6 | P | | | |
| | Gly-Phe-Phe-Leu-DNM | 2.2 | | |
| | Tyr-$NH_2$ | 1.0 | 2 | a/PRM |
| 7 | P—Gly-Phe-Phe-Leu-PRM | 1.3 | | b/galactosamine |
| | Gly-Phe-Phe-Leu-galactosamine | 0.9 | | |
| | Tyr-$NH_2$ | 1.0 | 3 | DNM |
| 8 | P | | | |
| | Gly-Phe-Leu-Gly-DNM | 3.0 | | |
| | Tyr-$NH_2$ | 1.0 | 3 | PRM |
| 9 | P | | | |
| | Gly-Phe-Leu-Gly-PRM | 3.2 | | |
| | Tyr-$NH_2$ | 1.0 | 3 | a/PRM |
| 10 | P—Gly-Phe-Leu-Gly-PRM | 2.3 | | b/galactosamine |
| | Gly-Phe-Leu-Gly-galactosamine | 1.8 | | |
| | Tyr-$NH_2$ | 1.0 | 4 | a/DNM |
| 11 | P—Gly-Phe-Leu-Gly-DNM | 3.5 | | b/galactosamine |
| | Gly-Phe-Leu-Gly-galactosamine | 2.0 | | |
| | Tyr-$NH_2$ | 1.0 | 4 | a/DNM |
| 12 | P—Gly-Phe-Leu-Gly-DNM | 2.2 | | b/fucosylamine |
| | Gly-Phe-Leu-Gly-fucosylamine | | | |

[a]DNM: daunomycin, PRM: puromycin.

### *Cleavage of HPMA copolymer-drug conjugates by lysosomal enzymes* in vitro

### *Enzymes*

Two sources of lysosomal enzymes were used: a mixture of rat liver lysosomal enzymes (tritosomes) prepared according to the method of Trouet (1974) and two purified lysosomal enzymes isolated from bovine spleen (Pohl *et al.*, 1982), cathepsin B (EC. 3.4.22.1) and cathepsin L (EC. 3.4.22.15).

The activity of the thiol-dependent proteinases in tritosomes was determined using the substrate Bz-Phe-Val-Arg-NAp. Release of *p*-nitroaniline was monitored under standard conditions as described by Šubr *et al.* (1986) ($\Delta A^{1\,cm} = 0.190$).

Activity of cathepsin B was determined using Bz-Arg-NAp. Enzyme (20 μl of a stock solution containing $8.1 \times 10^{-6}$ M active protein as determined by active site titration using iodacetic acid (Pohl *et al.*, 1982)) was incubated with Bz-Arg-NAp (25 μl, $9.2 \times 10^{-2}$ M in DMF), EDTA (1 mM) in citrate/phosphate buffer (935 μl, pH 5.5) and reduced glutathione (20 μl, 250 mM). The *p*-nitroaniline liberated was determined spectrophotometrically at 410 nm, $\Delta A^{1\,cm} = 0.280/10$ min.

Cathepsin L activity was determined using the substrate Bz-Phe-Val-Arg-NAp. Enzyme solution (20 μl of a 1.07 mg $ml^{-1}$ solution as determined using $A^{1\%\,1\,cm}_{280} = 11.4$, corresponding to $4.7 \times 10^{-6}$ M of active protein as determined by titration with 5,5′-dithiobis(2-nitrobenzoic acid), Pagano *et al.*, 1980), was incubated with reduced glutathione (250 mM 20 μl), citrate/phosphate buffer (940 μl pH 5.5), containing 1 mM EDTA, Bz-Phe-Val-Arg-NAp in DMF ($1.12 \times 10^{-2}$ M, 20 μl). The *p*-nitroaniline liberated was determined spectrophotometrically at 410 nm, $\Delta A^{1\,cm} = 0.830/10$ min.

### *Incubation of HPMA copolymer-drug conjugates with lysosomal enzymes*

Drug-polymer conjugates (3.8–7.2 mg $ml^{-1}$) were incubated at 37°C in 0.2 M citrate/phosphate buffer, pH 5.5, containing EDTA (1 mM) and reduced glutathione (5 mM), with tritosomes (0.48 ml $ml^{-1}$, plus 0.2% Triton X-100), with cathepsin L ($4.7 \times 10^{-7}$ M) or with cathepsin B ($8.1 \times 10^{-7}$ M). At various time intervals 0.1 ml of the incubation mixture was removed and free drug (daunomycin or puromycin) isolated by extraction into a mixture of 1 ml of 0.2 M $Na_2CO_3/NaHCO_3$ buffer (pH 9.8) and 1.5 ml of ethyl acetate. The organic layer was separated and the concentration of drug determined spectrophotometrically using the extinction coefficients given previously. Control experiments showed the efficiency of extraction was $100 \pm 5\%$ for daunomycin and $94.6 \pm 5\%$ for puromycin.

*Evaluation of HPMA copolymer-drug conjugates against L1210 cells grown* in vitro

L1210 cells were maintained in suspension culture (RPMI medium plus 10% heat-inactivated horse serum, 5% $CO_2$) using techniques previously described (Zenebergh *et al.*, 1982). Drug testing was carried out while cells were in a phase of exponential growth with a doubling time of 15–20 h. Cells were diluted into 10 ml of culture medium to give a starting cell density of ~10,000 cells $ml^{-1}$. They were cultured for 24 h addition of either free drug or drug conjugate at a range of concentrations. Each was then mixed thoroughly (including control cultures without addition) and cultured for 72 h. Cell numbers were measured at the beginning and the end of each experiment using a Coulter counter. Cell viability was assessed microscopically using trypan blue penetration as an indication of cell death.

In certain experiments, cells were harvested after 72 h exposure to drug or drug-conjugate and washed three times in phosphate-buffered saline (at room temperature). The final cell pellet was resuspended in warm (37°C) culture medium to give a concentration of 2,000–5,000 cells per ml. Aliquots (0.2 ml) of this solution were placed in a multiwell microtitre plate and the cells maintained at 37°C/5%$CO_2$ for the next ten days. Daily cell samples were taken from the microtitre plate, cell number counted and cell viability measured as described above.

*Binding and internalization of $^{125}$I-labelled conjugates by L1210 cells* in vitro

Tyrosinamide residues incorporated into the HPMA copolymers permitted polymer radioiodination using the chloramine T method (Duncan *et al.*, 1981). The radiolabelled polymers produced were stable during storage and experimentation and had a specific activity of ~25 $\mu$Ci $mg^{-1}$.

To measure binding and/or internalization of the polymer cells were incubated at 4°C or 37°C with radiolabelled HPMA copolymer for periods up to 72 h. At each sample time duplicate 1 ml samples of culture medium were taken, before removing excess culture medium from the cell pellet by washing the cells three times in PBS (room temperature). The final cell pellet was resuspended in 1 ml PBS and, with the two samples of culture medium, assayed for radioactivity. Coulter counting was used to assess the cell number present at the beginning and the end of the experiment.

Radioactivity binding to, or internalized by, the cells was expressed as the volume of culture medium ($\mu$l) whose contained substrate was bound/captured per $10^6$ cells (for further definition of units see Williams *et al.*, 1975).

### Results

*Release of daunomycin and puromycin from HPMA copolymer-conjugates during incubation with lysosomal enzymes* in vitro

Release of daunomycin or puromycin from HPMA copolymers by tritosomes or purified lysosomal enzymes, was more effective if the drug was attached to polymer via a Gly-Phe-Leu-Gly side-chain (Figure 2). In all cases the extent of daunomycin and puromycin released was similar during incubation of polymer with tritosomes or the purified enzyme cathepsin L (at the concentration used). Cathepsin B was however, less efficient in liberating daunomycin and marginally less effective in releasing puromycin. In all cases, control experiments carried out without addition of enzyme showed no drug liberation over three days (results not shown). The rate of drug cleavage was found to be independent of the galactosamine content of the polymer (results not shown).

During degradation of copolymer 10 by tritosomes to yield free puromycin, the first cleavage position was not between the terminal amino acid residue (glycine) and puromycin (results not shown). Sephadex G-15 chromatography of samples of incubation mixture taken at various time intervals showed elution of a low molecular weight puromycin derivative, either Gly-PRM or Leu-Gly-PRM. The broader elution profile of daunomycin on Sephadex G-15 did not permit discrimination between free drug and low molecular weight peptidyl derivatives but HPLC analysis indicated release of free daunomycin (results not shown).

*Effect of HPMA copolymer-drug conjugates on growth of L1210 and CCRF leukaemia* in vitro

HPMA copolymer-daunomycin conjugates (samples 5, 6 and 8) were all less effective than free daunomycin at inhibiting L1210 cell growth over 72 h (Figure 3). It can be seen that the inhibitory activity of a drug-conjugate is related to the amino acid composition of the drug-polymer linkage, Gly-Phe-Phe-Leu > Gly-Phe-Leu-Gly > Gly-Gly. In contrast with these daunomycin HPMA copolymers, which did show considerable inhibitory activity, the puromycin-containing copolymers (samples 7, 9 and 10) were virtually inactive against L1210 leukaemia over the concentration range investigated (Figure 4). Free puromycin did inhibit cell growth at these concentrations.

The effect of incorporation of the amino sugars, galactosamine and fucosylamine, into HPMA copolymers bearing daunomycin is shown in Figure 5. The presence of fucosylamine clearly enhanced the inhibitory effect of P-Gly-Phe-Leu-Gly-DNM, whereas incorporation of galactosamine did not. The human leukaemia, CCRF was found to be more sensitive to HPMA-daunomycin conjugates than L1210 leukaemia, but it can be seen (Figure 6) that here fucosylamine residues did not potentiate the activity of P-Gly-Phe-Leu-Gly-DNM.

*Evaluation of HPMA copolymer-daunomycin conjugate cytotoxicity against L1210 leukaemia*

To determine the concentration of HPMA-conjugate containing daunomycin needed to produce a cytotoxic effect, L1210 cells were incubated with samples 8 and 12 (drug concentrations of 16–184 $\mu g\, ml^{-1}$) for 72 h. Cells washed free of drug conjugate were plated out at low density and their growth curves (over 10 days) are shown in Figure 7. Although cells incubated with the lower concentrations of drug-conjugate did eventually show evidence of cell division, all treated cultures grew up more slowly than untreated cell cultures, and the cells exposed to the highest drug concentration (184 $\mu g\, ml^{-1}$) did not show any evidence of cell division within the experimental period.

*Accumulation of $^{125}$I-labelled HPMA copolymers by L1210 cells cultured* in vitro

L1210 cells were incubated with $^{125}$I-labelled polymers (samples 8, 10 and 11) for 3 h, either at 37°C or 4°C. Data shown in Figure 8(a) show the L1210 cell accumulation of radioactivity over 3 h. It can be seen that both the daunomycin-containing, and puromycin-containing copolymers became associated with the cells rapidly (even at time zero) and to a greater extent than an unsubstituted polymer. Over a 2 h incubation period there was little progressive accumulation of radioactivity at either

**Figure 2** Cleavage of polymer-drug conjugates by Tritosomes (a) and (d), cathepsin L, (b) and (e), and cathepsin B (c) and (f). Release of drug from the peptidyl side-chains GlyPheLeuGly-drug (●——●) and GlyPhePheLeu-drug (○——○) is shown. For daunomycin-containing polymers, (○——○) represents sample 6 and (●——●) sample 8. For puromycin (○——○) sample 7 and (●——●) sample 10. The incubation conditions are described in the **Methods**. The results shown are typical data, representative of at least two experiments in each case.

temperature. Further experiments showed that incorporation of both daunomycin and fucosylamine into HPMA copolymer structure greatly increased the cell association of copolymer. This was also apparently not followed by progressive accumulation of radioactivity by the cells over 3 h.

### Discussion

N-(2-Hydroxypropyl)methacrylamide copolymer-drug conjugates have been synthesized to contain little or no free drug (less than 0.1% relative to the drug bound). The solubility of daunomycin-containing copolymers in physiological buffers was limited, particularly above a conjugate concentration of 25 mg ml$^{-1}$, and this seemed to be a temperature-dependent phenomenon. Increasing temperature (from 20 to 30°C) sometimes caused precipitation. This process was found to be reversible (by lowering the temperature) and was probably due to formation of aggregates of polymer chains. These polymer–polymer (or polymer–protein) interactions are currently being investigated further (Ulbrich *et al.*, 1986). Problems of solubility were not observed during incubation of puromycin-containing polymers.

**Figure 3** Effect of oligopeptide spacer on the toxicity of polymer-daunomycin to L1210 cells cultured *in vitro*. Cells were cultured for 72 h in the presence of free daunomycin (□——□) or polymer-drug conjugates; polymer 5 (●——●) polymer 8 (○——○), polymer 6 (■——■). The points represent the mean (±s.e.) of at least three experimental values.

**Figure 5** Effect of incorporation of carbohydrate residues on the toxicity of polymer-daunomycin conjugates against L1210 cells cultured *in vitro*. Cells were incubated for 72 h with free daunomycin (●——●) or polymer-drug conjugates; polymer 8 (●– – –●), polymer 11 (▲——▲), polymer 12 (■——■). Points represent the mean (±s.e.) of at least three experimental values.

**Figure 4** Toxicity of polymer-puromycin against L1210 cells cultured *in vitro* cells were cultured for 72 h in the presence of free puromycin (●——●) or polymer-drug conjugates; polymer 7 (●– – –●), polymer 10 (○——○), polymer 9 (■——■).

**Figure 6** Effect of polymer-daunomycin conjugates on the growth of CCRF cells *in vitro*. Cells were incubated for 72 h in the presence of free daunomycin (●), or polymer conjugates; polymer 8 (○——○) and polymer 12 (●——●). Each point represents the mean (±s.e.) of at least three experimental values.

Greatest enzymatic release of drug (both daunomycin and puromycin) occurred when the peptide side-chain Gly-Phe-Leu-Gly was used as a polymer-drug spacer. Comparing the rate of drug release with that previously reported for liberation of terminal *p*-nitroaniline (NAp) residues from HPMA copolymers (Duncan *et al.*, 1983) shows that, for the same oligopeptide side-chain, the rate of cleavage was puromycin > NAp > daunomycin. This must reflect the specificities of the substrate-enzyme active site interactions, as discussed in detail by Kopeček (1984). It was also found (results not shown) that HPMA copolymer-drug conjugates bearing both daunomycin and carbohydrate residues (e.g. polymer 11) were cleaved at identical rates to those without sugar moieties, indicating that neighbouring carbohydrate residues did not effect enzyme access or specificity. Trouet *et al.* (1982) have also shown that biodegradable peptidyl spacers may be used as drug-carrier linkages. They bound daunomycin to succinylated bovine serum albumin via amino acids (di-, tri- and tetra-peptides), and showed that the sequence Ala-Leu-Ala-Leu-DNM released 75% of the bound drug during a 10 h incubation with tritosomes. They also found the efficiency of drug release from albumin was directly related to the length of the oligopeptide spacer, an observation we have reported previously in relation to the hydrolysis of side-chains in HPMA copolymers (Kopeček *et al.*, 1981). However, degradation of HPMA copolymer side-chains is a complex process and is influenced by length, side-chain composition and terminal group.

**Figure 7** Colony forming ability of L1210 cells after previous exposure (72 h) to polymer-daunomycin conjugates. Cells were incubated in culture medium without addition (●——●) or in the presence of HPMA-daunomycin; sample 12, 0.25 mg ml$^{-1}$ (○——○), sample 8, 0.5 mg ml$^{-1}$ (△——△), sample 12, 0.5 mg ml$^{-1}$ (■——■) and samples 8 and 12 at a polymer-drug concentration of 2 mg ml$^{-1}$ (▲——▲). The polymer-drug concentrations given above represent daunomycin concentrations of 184 $\mu$g ml$^{-1}$ for sample 8 (2 mg ml$^{-1}$) and 130 $\mu$g ml$^{-1}$ for sample 12 (2 mg ml$^{-1}$). Note that these cell cultures reach a maximum tolerated cell density ($\sim 8 \times 10^5$) above which the culture degenerates.

**Figure 8** Cell association of $^{125}$I-labelled HPMA copolymer-drug conjugates incubated *in vitro* with L1210 cells. (a) Radiolabelled copolymer, samples 8, 10 and 11 and an unsubstituted control polymer (all at 250 $\mu$g ml$^{-1}$) were incubated with L1210 cells for 0 min, [open bar] or 3 h at 4°C [hatched bar] or 37°C [solid bar] Cell association with radioactivity at the end of the incubation period is shown, mean ±s.e. in terms of $\mu$l of culture medium whose contained substrate becomes associated with 10$^6$ cells. (b) Cell association of polymer-daunomycin conjugates (37°C) containing; no other substitutent [hatched bar] sample 8, galactosamine [solid bar] sample 11, or fucosylamine [open bar] sample 12, are compared. The mean cell association (±s.e.) is shown.

For many years it has been postulated that the anthracycline antibiotics, daunomycin and adriamycin, interfere with cell division by intercalating into the DNA double-helix and therefore prevent normal mitosis. However, more recently it has become apparent that the anthracyclines can also interact with the cell membranes and induce detrimental effects from the outside of the cell (Tritton & Yee, 1982; Tokes *et al.*, 1982). In this study free daunomycin was shown to be very effective at inhibiting L1210 cell growth (Figure 3). Polymer-daunomycin conjugates were also able to prevent growth, in a dose-dependent manner (Figure 3) and this effectiveness varied with the oligopeptide spacer chosen to join polymer and drug. In this study the relative order of activity of drug conjugates against L1210 cells was P-Gly-Phe-Phe-Leu-DNM > P-Gly-Phe-Leu-Gly-DNM > P-Gly-Gly-DNM. Previously we have shown, using P-Gly-Gly[$^3$H]DNM, that the glycylglycyl spacer is not degradable by lysosomal enzymes (Kopeček & Duncan, 1986) and this may account for its limited activity. Cell-surface activity of this conjugate would explain the growth-inhibiting activity observed. Both drug-conjugates containing oligopeptide sequences known to be degradable by lysosomal enzymes (Figure 2) were found to be more effective, but it is perhaps surprising that polymer 6 displayed greater activity than polymer 8, as the former gave a slower rate of daunomycin release on incubation with rat and bovine lysosomal enzymes (Figure 2). This apparent discrepancy could represent a difference between the L1210 lysosomal enzymes and the other lysosomal enzymes studied, or alternatively may indicate increased daunomycin-membrane interactions due to the hydrophobic sequence Phe-Phe-Leu of polymer 6 side-chains. The latter could increase membrane-induced toxicity. Data shown in Figure 8 confirm that all the radioiodinated HPMA copolymers investigated containing daunomycin have a much greater affinity for L1210 cells than similar polymers without this drug. It is important to note that several different preparations of HPMA-daunomycin were used (samples 5, 8 and 12) and they showed reproducible, dose-dependent toxicity.

In contrast, the puromycin-containing conjugates were much less active against L1210. Since free puromycin did inhibit growth over the same time-period, it must be concluded that the L1210 cells were unable to release puromycin from the drug-conjugate (as the rat and other lysosomal enzymes did; Figure 2), that puromycin was released intracellularly in an inactive form or the cells did not accumulate the macromolecular drug quickly enough to ensure the required intracellular concentration.

Cell-surface receptors that recognize carbohydrate residues on macromolecules can, in theory, be used to promote more rapid cellular uptake of drug-conjugates (Duncan, 1986). Utilization of the known receptor on L1210 cell membranes recognizing fucose residues (Monsigny *et al.*, 1984) enabled the design of the HPMA copolymer-daunomycin conjugate (polymer 12) with increased activity against the mouse leukaemia *in vitro* (Figure 5). A similar drug-conjugate containing galactosamine (substituted at approximately the same mol%) did not differ significantly in activity from the non-carbohydrate-containing, polymer 11. This observation demonstrates the possibility of increasing the therapeutic index of a macromolecular drug-carrier by increasing the rate of capture by particular target cells. However, observation that the human T-cell lymphoblastoid leukaemia CCRF did not show the same specificity regarding the fucose-containing polymer (Figure 6) illustrates the difficulty in extrapolating any potentially useful targeting system from one cell type or another. It is interesting to note that the human leukaemia was more sensitive to both free and polymer-bound daunomycin than L1210, and indeed the maximum growth-inhibiting effects for CCRF (shown in Figure 6) were greater than those found for any polymer-drug conjugate against L1210 cells.

Investigations into the mechanism of action of HPMA-drug conjugates are continuing. At this stage we can confirm, using the analysis reported earlier, HPLC analysis of the preparations, and the measured activities of daunomycin–HPMA conjugates against L1210 and CCRF cells *in vitro* are not due to the presence of free drug in the conjugate. This contrasts with observations of Van Heeswijk *et al.*, (1984), who prepared polyglutamic acid–adriamycin conjugates, tested using an L1210 clonogenic assay and reported cytotoxic activity. Since these conjugates contained 0.5–2% weight free adriamycin, their cytotoxicity was largely ascribed to free adriamycin.

In all cases maximum inhibition of cell growth by polymer–drug was between 10–20% (Figures 3–5) and this can be explained by the nature of assay. Measurement of radiotracer incorporation (e.g. [$^3$H]thymidine) would be expected to register zero in respect of a control valve over the same incubation periods. To determine whether anti-cancer agents are lethal to tumour cells or simply cause a transient retardation of cell division it is important to perform clonogenic assays (Roper & Drewinko, 1976). Concentrations of daunomycin conjugate can be employed that are efficiently cytotoxic: no cell regeneration was observed over the 10 days subsequent to exposure of L1210 cells to the highest concentrations of polymers 8 and 12. The ability of daunomycin-HPMA copolymer conjugates to destroy L1210 cells has recently been demonstrated *in vivo* (Duncan *et al.*, 1986). HPMA-daunomycin conjugates administered intraperitoneally to $DBA_2$ mice (daunomycin concentration $5\,mg\,kg^{-1}$) prolong the survival time (and in the cases of optimum treatment produce long-term survivors (>50 days)), of individuals previously inoculated intraperitoneally with $10^5$ L1210 cells.

This study shows that polymer-drug conjugates can be synthesized that display many of the necessary features that could allow controlled delivery of anticancer agents *in vivo*. The conjugates contain little or no free drug; drug is only liberated by specific enzymatic hydrolysis; conjugates display cytotoxic activity against mouse and human leukaemia *in vitro*; activity is related to the sequence of the oligopeptide spacer used to attach drug to polymer; and activity can be potentiated by incorporation of residues (such as fucosylamine) into the polymer structure that target to specific cell-membrane receptors. Further experiments are in progress to characterize the pharmacokinetics of these conjugates *in vitro* and determine their therapeutic index against model tumours *in vivo*.

We would like to thank the Cancer Research Campaign for funding this work and The Royal Society for supporting the international collaboration. R.D. would like to thank Dr M. Fox, Paterson Institute for Cancer Research, Christie Hospital & Holt Radium Institute, Manchester, UK for helpful advice on L1210 cell culture techniques, also Lynne Scarlett for excellent technical assistance.

### References

ARNON, R. (1982). Antibodies and dextran as an anti-tumour drug carrier. In *Targeting of Drugs*, Gregoriadis *et al.* (eds) p. 31. Plenum Press: New York.

CARTLIDGE, S.A., DUNCAN, R., LLOYD, J.B., KOPEČKOVÁ-REJMANOVÁ, P. & KOPEČEK, J. (1986). Soluble, crosslinked N-(2-hydroxypropyl)methacrylamide copolymers as potential drug carriers. 3. Targeting by incorporation of galactosamine residues. Effect of route of administration. *J. Controlled Release* (in press).

CHENG, P.W. & BOAT, T.F. (1978). An improved method for the determination of galactosaminitol, glucosaminitol, glucosamine and galactosamine on an amino acid analyzer. *Anal. Biochem.*, **85**, 276.

DUNCAN, R., REJMANOVÁ, P., KOPEČEK, J. & LLOYD, J.B. (1981). Pinocytic uptake and intracellular degradation of N-(2-hydroxypropyl)methacrylamide copolymers. A potential drug delivery system. *Biochim. Biophys. Acta*, **678**, 143.

DUNCAN, R., CABLE, H.C., LLOYD, J.B., REJMANOVÁ, P. & KOPEČEK, J. (1983). Polymers containing enzymatically degradable bonds, 7. Design of oligopeptide side-chains in poly N-(2-hydroxypropyl)methacrylamide copolymers to promote efficient degradation by lysosomal enzymes. *Makromol. Chem.*, **184**, 1997.

DUNCAN, R., KOPEČEK, J., REJMANOVÁ, P. & LLOYD, J.B. (1983*a*). Targeting of N-(2-hydroxypropyl)methacrylamide copolymers to liver by incorporation of galactose residues. *Biochim. Biophys. Acta*, **755**, 518.

DUNCAN, R., CABLE, H.C., REJMANOVÁ, P., KOPEČEK, J. & LLOYD, J.B. (1984). Tyrosinamide residues enhance pinocytic capture of N-(2-hydroxypropyl)methacrylamide copolymers. *Biochim. Biophys. Acta*, **799**, 1.

DUNCAN, R., LLOYD, J.B., REJMANOVÁ, P. & KOPEČEK, J. (1985). Methods of targeting N-(2-hydroxypropyl)methacrylamide copolymers to particular cell types. *Makromol. Chem.* Suppl., **9**, 3.

DUNCAN, R., SEYMOUR, L.C.W., SCARLETT, L. LLOYD, J.B., REJMANOVÁ, P. & KOPEČEK, J. (1986). Fate of N-(2-hydroxypropyl)methacrylamide copolymers with pendent galactosamine residues after intravenous administration to rats. *Biochim. Biophys. Acta*, **880**, 62.

DUNCAN, R. (1986). Selective endocytosis of macromolecular drug carriers. In *Sustained and Controlled Release Drug Delivery Systems*, Lee, V.H.L. & Robinson, J.R. (eds). Marcel Dekker: New York (in press).

GARNETT, M.C., EMBLETON, M.J., JACOBS, E. & BALDWIN, R.W. (1985). Studies on the mechanism of action of an antibody-targeting drug carrier conjugate. *Anti-cancer Drug Design*, **1**, 3.

GERAN, R.I., GREENBERG, N.H., MACDONALD, M.M., SCHUMACHER, A.M. & ABBOTT, B.J. (1972). Protocols for screening chemical agents and natural products against animal tumours and other biological systems. *Cancer Chemotherap. Reps.*, **3**, 1.

GREGORIADIS, G. (1981). Targeting of drugs: implications in medicine. *Lancet*, **ii**, 241.

HOES, C.J.T., POTMAN, W., VAN HEESWIJK, W.A.R., MUD, J., DEGROOTH, B.G., GREVE, J. & FEIJEN, J. (1985). Optimization of macromolecular prodrugs of the antitumour antibiotic adriamycin. *J. Controlled Release*, **2**, 205.

KATO, T. (1983). Encapsulated drugs in targeted cancer therapy. In *Controlled Drug Delivery Vol. II*, Bruck, S.D. (ed) p. 189. CRC Press: Boca Raton, Florida.

KOPEČEK, J. (1977). Reactive copolymers of N-(2-hydroxypropyl)-methacrylamide with N-methacryloylated derivatives of L-leucine and L-phenylalanine. I. Preparation characterization and reaction with diamines. *Makromol. Chem.*, **178**, 2169.

KOPEČEK, J. & REJMANOVÁ, P. (1979). Reactive copolymers of N-(2-hydroxypropyl)methacrylamide with N-methacryloylated derivatives of L-leucine and L-phenylalanine. II. Reaction with the polymeric amine and stability of crosslinks towards chymotryps *in vitro*. *J. Polym. Sci. Symp.*, **66**, 15.

KOPEČEK, J., REJMANOVÁ, P. & CHYTRY, V. (1981). Polymers containing enzymatically degradable bonds. 1. Chymotrypsin catalysed hydrolysis of p-nitroanilides of phenylalanine and tyrosine attached to side chains of copolymers of N-(2-hydroxypropyl)methacrylamide. *Makromol. Chem.*, **182**, 799.

KOPEČEK, J. (1984). Controlled biodegradability of polymers – a key to drug delivery systems. *Biomaterials*, **5**, 19.

KOPEČEK, J., REJMANOVÁ, P., STROHALM, J., ULBRICH, K., ŘÍHOVÁ, B., ČHYTRY, V., DUNCAN, R. & LLOYD, J.B. (1985*a*). Synthetic polymeric drugs. *British patent Appl.*, 8 500 209 (4.1.85).

KOPEČEK, J., REJMANOVÁ, P., DUNCAN, R. & LLOYD, J.B. (1985*b*). Controlled release of drug model from N-(2-hydroxypropyl)methacrylamide copolymers. *Ann. N.Y. Acad. Sci.*, **446**, 93.

KOPEČEK, J. & DUNCAN, R. (1986). Poly N-(2-hydroxypropyl) methacrylamide macromolecules as drug carrier systems. In *Controlled Release of Drugs from Polymeric Particles and Macromolecules*, Illum, L. & Davis, S.S. (eds). Adam Hilger: Bristol, UK (in press).

MONSIGNY, M., ROCHE, A.-C. & MIDOUX, P. (1984). Uptake of neoglycoproteins via membrane lectin(s) of L1210 cells evidenced by quantitative flow cytofluorometry and drug targeting. *Biol. Cell*, **51**, 187.

PAGANO, M., ENGLER, R., GELIN, M. & JAYLE, M.F. (1980). Kinetic study of the interaction between rat haptoglobin and rat liver cathepsin B. *Can. J. Biochem.*, **58**, 410.

PLUMMER, JR. T.H. (1976). A simplified method for determination of amino sugars in glycoproteins. *Anal. Biochem.*, **73**, 532.

POHL, J., BAUDYŠ, M., TOMASEK, V. & KOSTKA, V. (1982). Identification of the active site cysteine and of the disulphide bonds in the N-terminal part of the molecule of bovine spleen cathepsin B. *Febs Lett.*, **142**, 23.

POSTE, G. (1983). Liposome targeting *in vivo:* Problems and opportunities. *Biol. Cell*, **47**, 19.

REJMANOVÁ, P., LABSKY, J. & KOPEČEK, J. (1977). Aminolyses of monomeric and polymeric p-nitrophenyl esters of methacryloylated amino acids. *Makromol. Chem.*, **178**, 2159.

REJMANOVÁ, P., KOPEČEK, J., POHL, J., BAUDYŠ, M. & KOSTKA, V. (1983). Polymers containing enzymatically degradable bonds. 8. Degradation of oligopeptide sequences in N-(2-hydroxypropyl)methacrylamide copolymers by bovine spleen cathepsin B. *Makromol. Chem.*, **184**, 2009.

REJMANOVÁ, P., KOPEČEK, J., DUNCAN, R. & LLOYD, J.B. (1985). Stability in rat plasma and serum of lysosomally degradable oligopeptide sequences in N-(2-hydroxypropyl)methacrylamide copolymers. *Biomaterials*, **6**, 45.

ŘÍHOVÁ, B. & KOPEČEK, J. (1985). Biological properties of targetable poly N-(2-hydroxypropyl)methacrylamide-antibody conjugates. *J. Controlled Release*, **2**, 289.

ŘÍHOVÁ, B., KOPEČEK, J., KOPEČKOVÁ-REJMANOVÁ, P., STROHALM, J., PLOCOVÁ, D. & SEMORADOVA, H. (1986). *J. Chromatogr. Biomed. Appl.* (in press).

ROPER, P.R. & DREWINKO, B. (1976). Comparison of *in vitro* methods to determine drug induced lethality. *Cancer Res.*, **36**, 2182.

SEYMOUR, L.W., DUNCAN, R., STROHALM, J. & KOPEČEK, J. (1986). Effect of molecular weight ($\bar{M}_w$) of N-(2-hydroxypropyl)methacrylamide copolymers on body distribution and ultimate excretion after subcutaneous intraperitoneal and intravenous administration to rats. *J. Biomed. Mater. Res.* (submitted).

STROHALM, J. & KOPEČEK, J. (1978). Poly N-(2-hydroxypropyl) methacrylamide. 4. Radical polymerisation. *Angew. Makromol. Chem.*, **70**, 109.

ŠUBR, V., DUNCAN, R. & KOPEČEK, J. (1986). Degradation of oligopeptide sequences connecting poly N-(2-hydroxypropyl)methacrylamide chains by lysosomal cysteine proteinases. *J. Bioactive Comp. Polymers*, **1**, 133.

TOKES, J.A., ROGERS, K.E. & REMBAUM, A. (1982). Synthesis of adriamycin coupled polygluturaldehyde microspheres and evaluation of their cytostatic activity, *Proc. Natl Acad. Sci.*, **79**, 2026.

TRITTON, T.R. & YEE, G. (1982). The anticancer agent adriamycin can be actively cytotoxic without entering cells. *Science*, 248.

TROUET, A. (1974). Isolation of rat liver lysosomal enzymes. *Methods Enzymol.*, **31**, 323.

TROUET, A., MASQUELIER, M., BAURAIN, R. & DEPREZ-DE CAMPENEERE. (1982). A covalent linkage between daunorubicin and proteins that is stable in serum and reversible by lysosomal hydrolases, as required for a lysosomotropic drug carrier conjugate: *In vitro* and *in vivo* studies. *Proc. Natl Acad. Sci.*, **79**, 626.

ULBRICH, K., KONAK, C., TUZAR, Z. & KOPEČEK, J. (1986). *Makromol. Chem.* (submitted).

VAN HEESWIJK, W.A.R., STOFFER, T., EENINK, M.J.D., POTMAN, W., VAN DER VIJGH, W.J.F., VAN DER POORT, J., PINEDO, H.M., LELIEVELD, P. & FEIJEN, J. (1984). Synthesis, characterisation and antitumour activity of macromolecular prodrugs of adriamycin. In *Recent Advances in Drug Delivery Systems*, Anderson, J.M. & Kim, S.W. (eds) p. 77. Plenum Press: New York.

WILLIAMS, K.E., KIDSTON, E.M., BECK, F. & LLOYD, J.B. (1975). Quantitative studies of pinocytosis. I. Kinetics of uptake of $^{125}$I-labelled polyvinylpyrrolidone) by rat yolk sac cultured *in vitro*. *J. Cell Biol.*, **64**, 113.

ZAHARKO, D.S., PRZYBYLSKI, M. & OLIVERO, V.T. (1979). Binding anticancer agents to carrier molecules. *Methods Cancer Res.*, **16**, 347.

ZENBERGH, A., BAURIN, R. & TROUET, A. (1982). Cellular pharmacokinetics of aclacinomycin A in cultured L1210 cells. Comparison with daunorubicin and doxorubicin. *Cancer Chemotherap. Pharmacol.*, **8**, 243.